# Untargeted Metabolomics To Ascertain Antibiotic Modes of Action

**DOI:** 10.1128/AAC.02109-15

**Published:** 2016-03-25

**Authors:** Isabel M. Vincent, David E. Ehmann, Scott D. Mills, Manos Perros, Michael P. Barrett

**Affiliations:** a University of Glasgow, Glasgow, United Kingdom; b AstraZeneca, Waltham, Massachusetts, USA

## Abstract

Deciphering the mode of action (MOA) of new antibiotics discovered through phenotypic screening is of increasing importance. Metabolomics offers a potentially rapid and cost-effective means of identifying modes of action of drugs whose effects are mediated through changes in metabolism. Metabolomics techniques also collect data on off-target effects and drug modifications. Here, we present data from an untargeted liquid chromatography-mass spectrometry approach to identify the modes of action of eight compounds: 1-[3-fluoro-4-(5-methyl-2,4-dioxo-pyrimidin-1-yl)phenyl]-3-[2-(trifluoromethyl)phenyl]urea (AZ1), 2-(cyclobutylmethoxy)-5′-deoxyadenosine, triclosan, fosmidomycin, CHIR-090, carbonyl cyanide *m*-chlorophenylhydrazone (CCCP), 5-chloro-2-(methylsulfonyl)-*N*-(1,3-thiazol-2-yl)-4-pyrimidinecarboxamide (AZ7), and ceftazidime. Data analysts were blind to the compound identities but managed to identify the target as thymidylate kinase for AZ1, isoprenoid biosynthesis for fosmidomycin, acyl-transferase for CHIR-090, and DNA metabolism for 2-(cyclobutylmethoxy)-5′-deoxyadenosine. Changes to cell wall metabolites were seen in ceftazidime treatments, although other changes, presumably relating to off-target effects, dominated spectral outputs in the untargeted approach. Drugs which do not work through metabolic pathways, such as the proton carrier CCCP, have no discernible impact on the metabolome. The untargeted metabolomics approach also revealed modifications to two compounds, namely, fosmidomycin and AZ7. An untreated control was also analyzed, and changes to the metabolome were seen over 4 h, highlighting the necessity for careful controls in these types of studies. Metabolomics is a useful tool in the analysis of drug modes of action and can complement other technologies already in use.

## INTRODUCTION

There is a pressing need for new classes of antibiotics as resistance to those in use becomes ever more widespread (1).

Prior to the broad implementation of natural-product antibiotics, efforts had focused on the identification of small molecules that inhibit microbial metabolism. The sulfonamides, for example, target folate biosynthesis. The success of natural-product antibiotics (that hit targets such as DNA, RNA, cell wall, and protein synthesis [2]) served to limit exploration of the small-molecule space suitable for antibiotics. The hope for rational target-based discovery drove a burst of activity, leading to an era of screening chemicals for inhibitory activity against protein targets. However, this approach uncovered compounds too far from pharmacological utility to succeed in development (3). Because of this, as well as diminishing economic returns in antibiotic discovery, the antibiotic discovery pipeline has diminished (4).

The history of antimicrobial development led to a quest for useful drug targets that ended prematurely, and large areas of microbial metabolism have yet to be targeted using small-molecule chemicals. In the meantime, phenotypic screening, in which chemical libraries are screened for activity against whole microorganisms, is successfully identifying new antimicrobials. Novel targets suitable for drug development are appearing as a result of these studies (5–7). An advantage of phenotypic screening over the target-based screens that became popular in the 1980s to 2000s is that effective compounds are already endowed with key pharmacological attributes, such as selectivity for the microbe of interest, microbial membrane permeability, and chemical stability (or, conversely, the ability of prodrug to be metabolized to active compound), when identified. The phenotypic screening approach, however, fails to reveal the targets of these new compounds. This, in turn, can slow the process of lead optimization for lack of clarity in understanding how to improve on-target pharmacology.

There have been efforts to develop high-throughput screens to elucidate drug targets, including metabolic suppression and rescue (8), chemical probe synergism (9), and cytological profiling (10), but these methods have yet to be widely adopted and applied.

Metabolomics aims to identify all small-molecule metabolites in a given system, and the current technologies make use of nuclear magnetic resonance (NMR) analyses for highly abundant metabolites (11, 12) or high-resolution mass spectrometry coupled to sophisticated chromatography for more global analyses (13).

Metabolomic techniques are increasing in popularity in the hunt for biomarkers of disease (14, 15) and biomarkers of toxicity (16) and in drug mode-of-action studies (13, 17, 18). Ultimately, metabolomics takes direct, *in situ*, observations of drug inhibition of enzymes, through measurements of the metabolic substrates and products of these enzymes. In some instances, drugs act without inhibiting specific metabolic enzymes, but signatures related to inhibited macromolecules or other pathways may be acquired. In other cases, drugs are modified within the organism and mass spectrometry-based metabolomic approaches enable determination of drug metabolism as well (13, 19).

One study using metabolomics to analyze the mode of action (MOA) of drugs used eflornithine, an ornithine decarboxylase inhibitor, and highlighted the simplicity of using metabolomics to detect an increase in the substrate of the inhibited enzyme (in this case ornithine) and a decrease in the product (putrescine), allowing the single enzyme drug target to be pinpointed with precision (13). Mass spectrometry-based metabolomics has also been used to a limited extent to determine the modes of action of individual antibacterial compounds in bacteria (20–23), but its use in higher-throughput assays has not been determined.

The aim of this study was to determine whether metabolomics can be used systematically to determine the mode of action of antibiotics.

## MATERIALS AND METHODS

Escherichia coli strain W3110 Δ*tolC*::Tn*10* was used for all compound testing. HEK 293T (human embryonic kidney cell strain 293T) was used to test AZ7.

### *In vitro* susceptibility test methods.

For E. coli, compounds were dissolved to 6.4 mg/ml in dimethyl sulfoxide (DMSO). Compounds were then serially diluted in 100 μl cation-adjusted Mueller-Hinton broth (MHB) in a 96-well plate, and 100 μl cell suspension (7.5 × 10^5^ CFU/ml) was added, along with additives if required. These cultures were left in a static incubator for 24 h. The optical density at 590 nm (OD_590_) was read without shaking. Ceftazidime was used as a control. The MIC was then calculated.

For human embryonic kidney cells, Dulbecco's modified Eagle's medium with 10% fetal calf serum (DMEM-FCS) was aspirated from a 10-ml culture of confluent HEK 293T cells, and 2 ml of 0.25% trypsin-EDTA solution was added. Cells were incubated for 5 min before the cell suspension was collected in 8 ml of DMEM-FCS. Cells were centrifuged at 1,200 rpm and resuspended in 10 ml DMEM-FCS. Cells were counted, and a suspension was prepared at 3 × 10^5^ cells/ml. One hundred microliters of this suspension was added to each well of a 96-well plate, and cells were incubated for 3 h. Drug stocks were prepared at 128 μg/ml and serially diluted in a 96-well plate. One hundred microliters of each dilution was added to the corresponding well in the cell culture plate, and this plate was incubated for 16 h. Twenty microliters of a 12.5-mg/ml concentration of resazurin in phosphate-buffered saline (PBS) was added to each well of the 96-well plate, and plates were incubated for a further 24 h. The fluorescence of the resazurin was measured on an Optima Fluostar plate reader.

### Metabolite extraction from E. coli.

A 10-ml overnight culture of E. coli was inoculated into 40 ml of cation-adjusted MHB (no preincubation) or a 2-ml overnight culture of E. coli was inoculated into 48 ml of cation-adjusted MHB (with preincubation) and the mixture was incubated with shaking at 37°C. For assays without a preincubation step, the extractions were started at this point; for assays with a preincubation step, the cells were incubated for 2 h before extractions were started. Drugs were added at 4× MIC, and samples were taken at 0, 2, and 4 h after drug addition. Samples were cooled to 5°C in a dry-ice–ethanol bath to rapidly quench metabolism before they were transferred to ice. Ten milliliters of cells was pelleted at 3,000 relative centrifugal force (RCF), washed in 10 ml cold 0.85% NaCl, and then resuspended in 1 ml 0.85% NaCl. The OD_590_ of this cell suspension was taken and adjusted to 1. One milliliter of cells was pelleted and resuspended in 200 μl chloroform-methanol-water (1:3:1, by volume) (plus theophylline, 5-fluorouridine, *N*-methylglucamine, canavanine, and piperazine, all at 1 μM as internal standards). Samples were subjected to four freeze-thaw cycles in the dry-ice–ethanol bath with regular vortexing before a final centrifugation at 16,000 RCF. The supernatant was taken and kept at −80°C under argon.

### Metabolite extraction from human embryonic kidney cells.

Medium was aspirated from a 10-ml culture of confluent HEK 293T cells, and 2 ml of 0.25% trypsin-EDTA solution was added. Cells were incubated for 5 min before the cell suspension was collected in 8 ml of DMEM-FCS. Cells were centrifuged at 1,200 rpm and resuspended in 10 ml DMEM-FCS. Two milliliters of this cell suspension was added to 18 ml DMEM-FCS. Three milliliters of this cell suspension was seeded into each of six wells (34.8-mm well diameter). These wells were incubated for 48 h at 37°C, 5% CO_2_. AZ7 or DMSO solutions were prepared in 10 ml DMEM-FCS at 4× 90% inhibitory concentration (IC_90_) (AZ7) or an equal volume of DMSO. Culture medium was aspirated from the cultured cells, and 3 ml of drug-DMSO solution was added. This mixture was incubated for 4 h. Samples were cooled to 5°C in a dry-ice–ethanol bath before they were transferred to ice and pelleted at 3,000 RCF, transferred to a 1-ml Eppendorf tube, and washed in 1 ml 0.85% NaCl. Cells were pelleted again, the supernatant was removed, and cells were resuspended in 200 μl chloroform-methanol-water (1:3:1, by volume) (plus theophylline, 5-fluorouridine, *N*-methylglucamine, canavanine, and piperazine, all at 1 μM as internal standards). Samples were shaken for 1 h at 4°C before a final centrifugation at 16,000 RCF. The supernatant was taken and kept at −80°C under argon.

### Lipid extraction from E. coli.

Overnight cultures of E. coli were inoculated into cation-adjusted MHB and incubated with shaking at 37°C. A preincubation step of 2 h preceded drug addition. CHIR-090 or DMSO was added at 4× MIC, and samples were taken at 0, 2, and 4 h after drug addition. Samples were cooled to 5°C in a dry-ice–ethanol bath before they were transferred to ice. Ten milliliters of cells was pelleted at 3,000 RCF, washed in 10 ml cold 0.85% NaCl, and then resuspended in 1 ml 0.85% NaCl. The OD_590_ of this cell suspension was taken and adjusted to 1. One milliliter of cells was pelleted and moved in a minimal volume of NaCl to a glass vial to which 400 μl 2:1 chloroform-methanol by volume was added using a glass pipette. Samples were shaken for 10 min at room temperature, and 125 μl NaCl was added. Samples were vortexed and then left at room temperature for 20 min. The bottom layer was removed and placed in a glass vial to be stored under argon gas at 4°C.

### Data acquisition.

A 10-μl aliquot of each sample was run in a randomized order on a ZIC-pHILIC (polymeric hydrophilic interaction chromatography) column (SeQuant) or a ZIC-HILIC (hydrophilic interaction chromatography) column (SeQuant) coupled to an Orbitrap mass spectrometer (Thermo Scientific) or an Orbitrap Q Exactive mass spectrometer (Thermo Scientific) according to previously published methods (13). Lipid analysis was done using a C_30_ column (3 μm, 3 by 150 mm) (Thermo Dionex) coupled to an Orbitrap Velos instrument using data-dependent fragmentation on the three most intense ions.

Fragmentation of pHILIC column-separated metabolites was performed in a data-dependent manner on the Q Exactive (Thermo Scientific) mass spectrometer, with the five most intense ions picked in a 4 *m/z* exclusion window and at a collision energy of 65. All other conditions were the same as previously reported (13).

### Metabolomics data analysis.

Data analysis was performed using the MzMatch (24) and IDEOM (25) software packages for untargeted analysis. The Thermo Scientific Xcalibur software package was used for targeted peak picking and fragmentation analysis.

According to the metabolomics standards initiative (MSI), metabolite identifications (MSI level 1) are given when more than one feature matches an authentic standard (i.e., mass and retention time) and annotations are made when matching to a metabolite is made by mass only (MSI level 2) (26). A mixture of 240 standards, covering a range of metabolic pathways, was run with each sample batch to allow metabolite “identifications” to be made (MSI level 1). For metabolites without an authentic standard metabolite, “annotations” (MSI level 2) were made. Identifications and annotations were made using the IDEOM software package.

Lipid analyses were conducted using LipidSearch software (Thermo Scientific).

## RESULTS AND DISCUSSION

Assays that are able to determine the mode of action of new antibiotics are needed to accompany renewed interest in phenotypic screening of chemical libraries for antimicrobial activity. Where chemicals hit enzymes involved in metabolic pathways, perturbation to those pathways can be detected using metabolomics technology. Here we set out to determine the ability of metabolomics to report on modes of action of antibiotics in a systematic manner.

**FIG 1:**
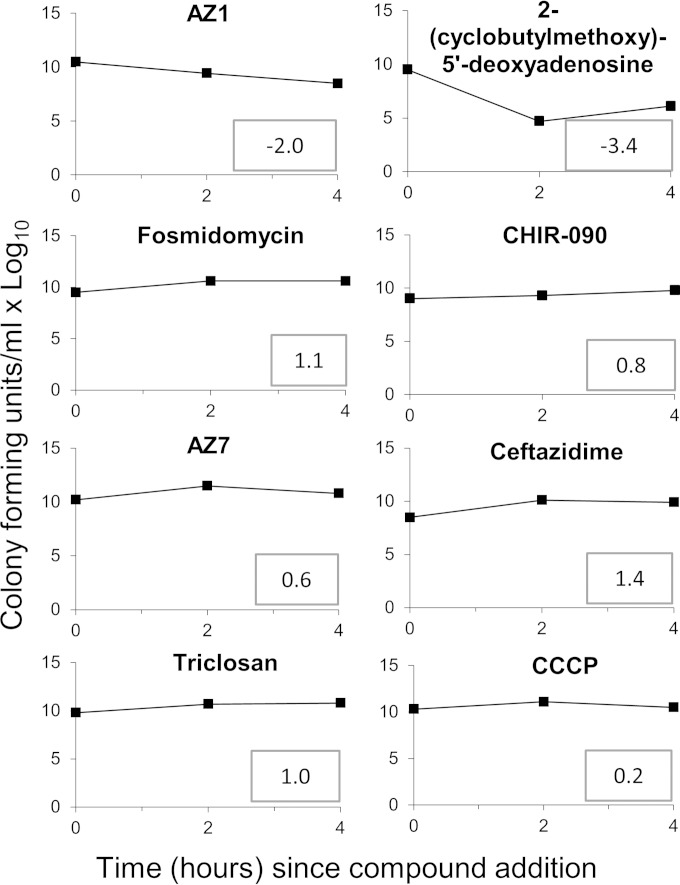
All test compounds had an effect on TolC^−^ E. coli within 4 h. The numbers of CFU at 4× MIC over a 4-h time course are shown. Numbers in boxes indicate the log_10_ increase after 4 h compared to the 0-h time point. The untreated cells showed a log_10_ increase of 2.3 after 4 h.

Eight compounds were tested with the Gram-negative organism Escherichia coli in our assay (Fig. 1). Six of these compounds had previously established tentative modes of action covering a range of targets, not all of which were metabolic (Table 1). One compound [5-chloro-2-(methylsulfonyl)-*N*-(1,3-thiazol-2-yl)-4-pyrimidinecarboxamide (AZ7)] had a completely unknown target. Researchers involved in data collection and analysis (I. M. Vincent and M. P. Barrett) were blind to the identity of the active compounds until after data analysis had been completed. The metabolomics method was compared to a radiolabel incorporation assay (27) that also aims to determine the mode of action of antibiotics. Using this method, the target of AZ1 was found to be broadly related to DNA metabolism and the target of triclosan was found to be broadly related to fatty acid metabolism, but the targets of the other compounds were mixed or no inhibition of the selected metabolic domains was seen (Table 1).

**TABLE 1:** Test compounds covering a range of metabolic areas^*a*^

Compound	Target	Radioactive assay result	MIC for ARC523 wild type (μg/ml)	MIC for ARC524 TolC^−^ (μg/ml)
1-[3-Fluoro-4-(5-methyl-2,4-dioxo-pyrimidin-1-yl)phenyl]-3-[2-(trifluoromethyl)phenyl] urea (AZ1)	Thymidylate kinase^*b*^	DNA	>64	0.0625–0.125
2-(Cyclobutylmethoxy)-5′-deoxyadenosine	DNA ligase^*c*^	Mixed	>64	2–8
Triclosan	Fatty acid metabolism^*d*^	Fatty acid	0.25	0.0039
Fosmidomycin	Deoxyxylulose 5-phosphate reductoisomerase^*e*^	No inhibition	2	2
CHIR-090	UDP-3-*O*-acyl-*N*-acetylglucosamine deacetylase (LpxC)^*f*^	Mixed	0.125	0.0078
CCCP	Oxidative phosphorylation	Mixed	32	0.5–1
5-Chloro-2-(methylsulfonyl)-*N*-(1,3-thiazol-2-yl)-4-pyrimidinecarboxamide (AZ7)	Unknown	Mixed	>64	16
Ceftazidime	Penicillin binding proteins^*g*^	Cell wall	0.25–0.5	0.25–0.5

a MICs of AZ compounds in wild-type E. coli ARC523 and the TolC mutant, strain ARC524. *n* ≥ 3. Where different values were found among the replicates, a range is shown.

b From reference 32.

c From reference 45 and M. Cavero-Tomas, M. Gowravaram, H. Huynh, H. Ni, and S. Stokes, PCT international patent application WO 2006040558 A1 20060420 (2006).

d From reference 46.

e From reference 47.

f From reference 35.

g From reference 2.

### MICs and kill kinetics.

The MICs of the compounds for wild-type E. coli strain ARC523 and a TolC^−^ derivative, ARC524, lacking the key TolC efflux pump were calculated (Table 1).

The TolC knockout strain was chosen for metabolomics experiments, as the wild-type strain was not inhibited by some of the compounds at our maximum dose of 64 μg/ml, whereas the TolC knockout strain often had lower MICs (this was considered to be likely due to drug efflux in the wild-type strain [28, 29]).

Kill kinetics were performed at 4× MIC at a high cell density (in order to provide enough material for metabolomics) and produced a growth defect after drug treatment compared to the result with untreated cells for all tested compounds (Fig. 1).

Other than those treated with 2-(cyclobutylmethoxy)-5′-deoxyadenosine, none of the compound-treated strains showed growth recovery after 4 h of treatment. We therefore used a 4-h protocol for our metabolomics protocol, with time points at 0, 2, and 4 h after treatment at 4× MIC, bearing in mind that a shorter time course would also be required for 2-(cyclobutylmethoxy)-5′-deoxyadenosine. For the primary analyses, pHILIC was used, creating the best separation of metabolites of central carbon metabolism, followed by Q Exactive mass spectrometry with fragmentation on a pooled sample. Hydrophilic interaction liquid chromatography (HILIC) and reversed-phase chromatography were used in secondary analyses where required, specifically to monitor polyamine metabolism or lipid metabolism, respectively. A standard mix with 240 standards was run with each batch of samples to provide level 1 metabolite identifications according to the metabolomics standards initiative (i.e., a match of retention time and mass to an authentic standard [26]). Between 70 and 77 of these metabolites were detected in our E. coli extracts by pHILIC and 67 by HILIC (variations between batches may be due to machine sensitivity variations, genuine differences in the levels of metabolites detected, or variability in the cell number producing unacceptable replicate variation). The remaining metabolites were annotated as level 2 annotations (a match of mass to an internal database [25] which includes all known E. coli metabolites).

### Metabolomics.

The number of metabolic features detected was between 850 and 2,655 base peaks and between 499 and 1,295 metabolite features for which a putative annotation could be given (see Data Sets S1 to S6 in the supplemental material), depending on the batch of samples run.

**FIG 2:**
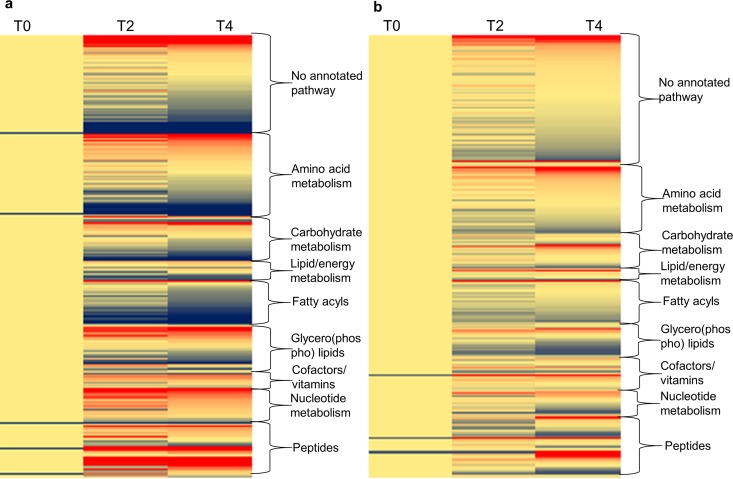
There is variation in the E. coli metabolome in the absence of drug. (A) No preincubation; (B) with a preincubation stage of 2 h. The scale is red to blue, with the darkest red being a >5-fold increase and the darkest blue being a >5-fold decrease in metabolite intensity. Yellow coloring represents no change to the metabolite intensity. Blue coloring in the time zero (T0) samples means that this metabolite was detected only after 2 and 4 h (T2 and T4). At least three biological replicates were run for each time point.

Substantial variation in the metabolome was evident over 4 h, even in the untreated control (Fig. 2), indicating the dynamic nature of metabolism under the growth conditions used. The experiment was initiated from a stationary-phase overnight culture either at a high inoculum without a preincubation step (Fig. 2a) or with a lower inoculum with a 2-h preincubation (Fig. 2b). There was less variation when the culture was preincubated with a lower inoculum (Fig. 2). This difference is likely due to the transition from stationary-phase planktonic growth to the exponential growth phase in the samples without preincubation, whereas the samples that were preincubated are in exponential growth phase over the entire time course.

The variation in the untreated, preincubated metabolome was most apparent in lipid metabolism, but there were also increases in the levels of some peptides, thymidine (C_10_H_14_N_2_O_5_, level 1 identification), and NAD^+^ (C_21_H_27_N_7_O_14_P_2_, level 2 annotation) (Table 2).

**TABLE 2:** Metabolite changes in untreated TolC^−^ E. coli over 4 h^*a*^

Formula	EcoCyc?	No. of Isomers	Possible pathway(s)	Fold change			*P* value by *t* test	
				T0	T2	T4	T2	T4
C_8_H_18_N_2_O_2_	Y	1	0	1	2.03	3.09	0.0212	1.03E-05
C_10_H_14_N_4_O_3_	Y	1	0	1	1.62	1.63	0.0007	0.0001
C_26_H_52_NO_7_P	N	12	0	1	0.69	0.44	0.1014	0.0002
C_6_H_5_N_5_O	Y	1	0	1	1.62	2.22	0.0499	0.0002
C_5_H_4_O_3_	N	4	0	1	7.63	8.61	0.0012	0.0007
C_4_H_3_NO_3_	N	1	0	1	1.41	2.46	0.0722	0.0009
C_18_H_34_O_2_	N	57	Fatty acid biosynthesis, biosynthesis of unsaturated fatty acids	1	0.73	0.56	0.2830	0.0004
C_18_H_30_O_4_	N	20	Fatty acids and conjugates	1	0.40	0.15	0.0068	0.0003
C_19_H_34_O_4_	N	3	Fatty acids and conjugates	1	0.63	0.34	0.1063	0.0007
C_19_H_36_O_2_	N	27	Fatty acids and conjugates	1	0.55	0.48	0.0106	0.0009
C_16_H_14_O_6_	N	33	Flavonoid biosynthesis	1	3.14	4.16	0.0816	0.0008
C_18_H_12_O_5_	N	2	Flavonoids	1	5.68	8.28	0.0034	1.34E-06
C_35_H_66_NO_8_P	N	8	Glycerophosphoethanolamines	1	0.39	0.18	0.0006	0.0001
C_23_H_44_NO_7_P	N	2	Glycerophosphoethanolamines	1	0.54	0.27	0.0053	0.0001
C_33_H_64_NO_8_P	N	7	Glycerophosphoethanolamines	1	0.49	0.25	0.0067	0.0003
C_35_H_68_NO_8_P	N	16	Glycerophosphoethanolamines	1	0.46	0.23	0.0032	0.0005
C_37_H_70_NO_8_P	N	20	Glycerophosphoethanolamines	1	0.29	0.10	0.0024	0.0010
C_41_H_77_O_10_P	N	14	Glycerophosphoglycerols	1	1.57	2.38	0.0058	0.0001
C_40_H_73_O_10_P	N	15	Glycerophosphoglycerols	1	0.33	0.07	0.0262	0.0001
C_42_H_77_O_10_P	N	18	Glycerophosphoglycerols	1	0.49	0.15	0.0086	0.0002
C_39_H_75_O_10_P	N	14	Glycerophosphoglycerols	1	1.35	2.21	0.1241	0.0007
C_27_H_49_O_12_P	N	1	Glycerophosphoinositols	1	0.59	0.16	0.1406	0.0005
C_40_H_78_NO_10_P	N	12	Glycerophosphoserines	1	0.79	0.46	0.0263	0.0002
C_35_H_62_O_4_	N	1	Hopanoids	1	0.36	0.15	0.0030	0.0009
C_18_H_30_N_4_O_6_	N	1	Hydrophobic peptide	1	7.26	9.92	0.0379	0.0004
C_18_H_31_N_5_O_6_	N	5	Hydrophobic peptide	1	2.03	3.61	0.0191	0.0007
C_17_H_28_N_4_O_8_	N	2	Hydrophobic peptide	1	1.64	1.60	0.0002	0.0008
C_16_H_24_	N	1	Isoprenoids	1	0.29	0.35	0.0005	0.0004
C_18_H_32_O_3_	N	40	Linoleic acid metabolism	1	0.67	0.39	0.0314	1.43E-05
C_18_H_32_O_3_	N	40	Linoleic acid metabolism	1	0.53	0.30	0.0387	0.0008
C_5_H_12_N_2_O	N	1	Lysine degradation	1	0.55	0.21	0.2320	0.0008
C_18_H_30_O_3_	N	38	Octadecanoids	1	0.50	0.21	0.0910	3.19E-05
C_21_H_27_N_7_O_14_P_2_	Y	1	Oxidative phosphorylation, glutamate metabolism, nicotinate and nicotinamide metabolism	1	2.02	2.00	0.0059	0.0008
C_10_H_14_N_2_O_5_	Y	1	Pyrimidine metabolism	1	2.48	2.32	0.0854	0.0001
C_19_H_39_NO_3_	Y	3	Sphingolipid metabolism	1	2.79	2.78	0.0001	0.0001
C_27_H_48_O_9_	N	1	Sterols	1	0.62	0.34	0.0748	0.0005
C_10_H_17_NO_6_	Y	2	Superpathway of linamarin and lotaustralin biosynthesis, linamarin biosynthesis, linamarin degradation	1	2.81	4.21	0.0032	0.0002
C_6_H_10_N_3_O_4_P	N	1	Thiamine metabolism	1	1.87	2.32	0.0012	0.0002

a Changes that are significant at a *P* of <0.001 after 4 h are shown. T0, T2, and T4, 0-, 2-, and 4-h time points. Formulae that match the EcoCyc database are indicated (Y, yes; N, no). Note that extensive data on lipids and peptides are missing from EcoCyc. C_18_H_30_N_4_O_6_ is included twice as it appears at two different retention times.

It was necessary to consider the inherent variation in the metabolome when analyzing the drug-induced changes, and this background variation was removed from all drug treatments.

To analyze the drug-induced changes to the metabolome, IDEOM (25), an Excel-based metabolite identification package, was used to sort metabolites by fold change and *P* value. Annotated metabolites (and their isomers) were mapped to KEGG pathways using Pathos, an open-source software available at http://motif.gla.ac.uk/Pathos (30). The shape of each peak associated with a feature appearing to change in abundance as a consequence of treatment was checked, and those with nonreproducible peaks or poor peak shapes (incomplete, shoulder peaks, multiple joined peaks, etc. [31]) were ruled out. Metabolites that were connected in pathways and behaved in similar ways over the time course were considered to be more likely identifications.

### Modes of action. (i) AZ1.

**FIG 3:**
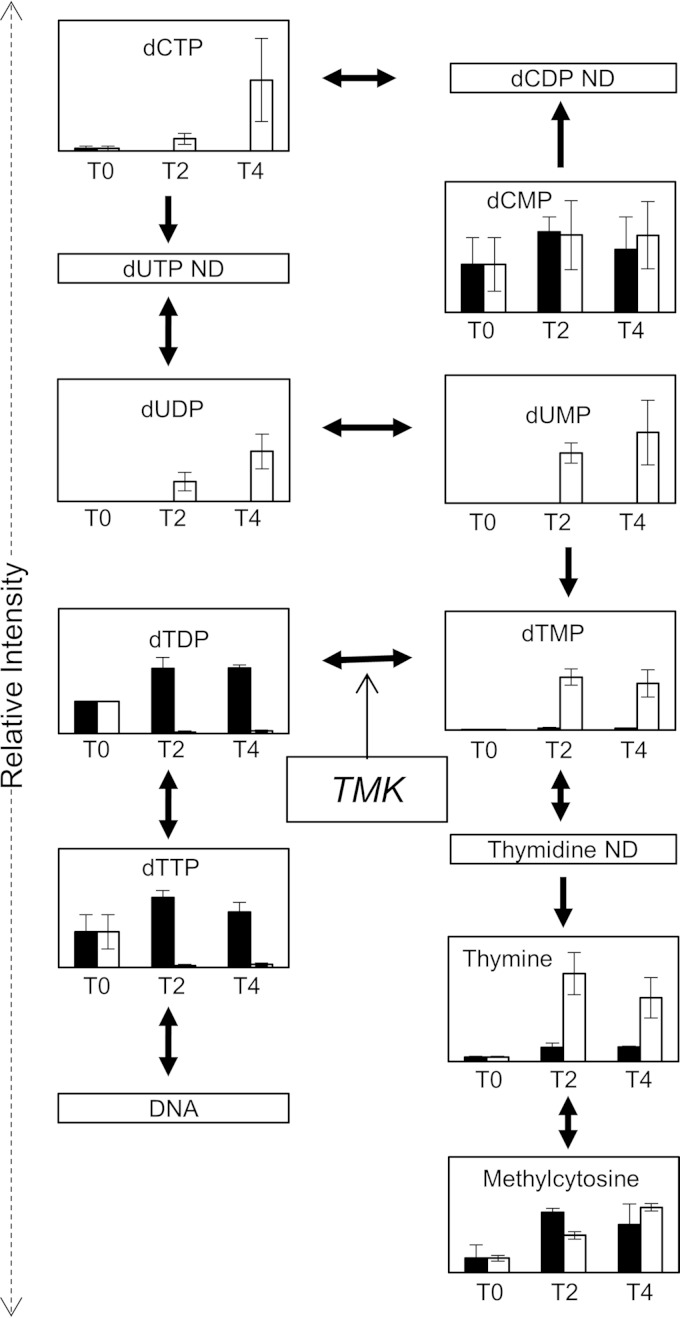
The mode of action of AZ1 was detectable by metabolomics. ND; not detected; TMK; thymidylate kinase. Black bars, no-drug control; white bars, treated with AZ1.The *y* axis shows metabolite intensity, and the *x* axis shows hours after drug addition. Error bars show standard deviations of the mean. ND, not detected.

The target enzyme for AZ1 was readily revealed using our metabolomics platform. AZ1 is an inhibitor of thymidylate kinase (32), and after treatment at 4× MIC, large increases in dTMP-related metabolites were seen alongside large decreases in dTDP-related metabolites (Fig. 3; see Fig. S1a and Data Set S1 in the supplemental material). There were also changes in the levels of annotated metabolites up and downstream of the target (dUMP, dUDP, and dCTP upstream of the target all increase, while dTTP, which is downstream of the target, decreases), but wider changes to the metabolome were not apparent. Thymidylate kinase is a target for drug discovery in a number of infectious diseases, including diseases caused by parasites, bacteria, and viruses (33). Our metabolomics platform may prove to be of use in whole-cell screening of potential thymidylate kinase inhibitors for use in these organisms.

### (ii) Fosmidomycin.

**FIG 4:**
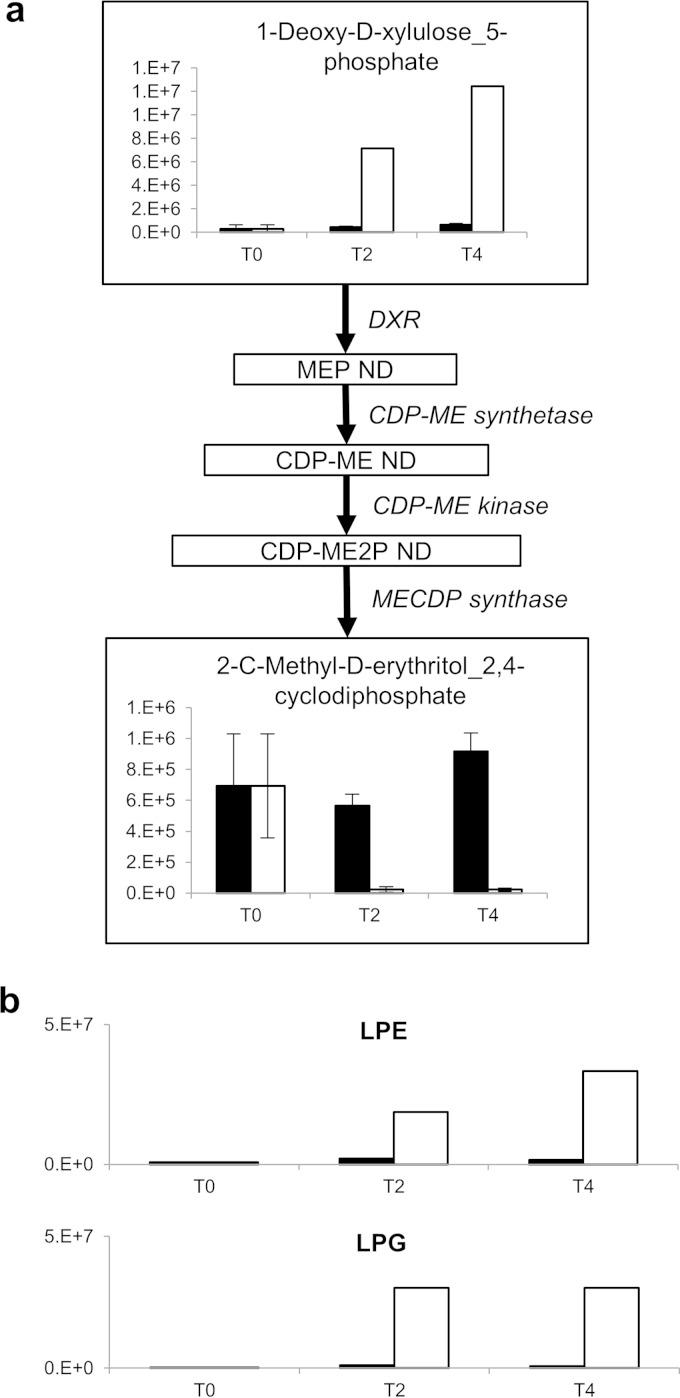
The modes of action of fosmidomycin (a) and CHIR-090 (b) were detectable by metabolomics. ND, not detected; DXR, DXP reductoisomerase; MEP, methylerythritol phosphate; ME, methylerythritol; ME2P, methylerythritol cyclodiphosphate. Black bars, no-drug control; white bars, drug treated. The *y* axis shows metabolite intensity, and the *x* axis shows hours after drug addition. Error bars show standard deviations of the mean.

Fosmidomycin clearly inhibited the DXR pathway in our blind analysis, due to large changes in 1-deoxy-d-xylulose 5-phosphate (DXP, annotated), 2C-methyl-d-erythritol 2,4-cyclodiphosphate (ME-CDP, annotated) (Fig. 4a; see Fig. S1b in the supplemental material), and metabolites related to DXP and ME-CDP (see Data Set S2 in the supplemental material). However, the individual target enzyme (deoxyxylulose reductase) was not precisely pinpointed, as some metabolites of the isoprenoid biosynthesis pathway were not detected (this may be due either to their concentrations being lower than the detection limit or suboptimal ionization). A targeted metabolomics approach has previously been used to analyze fosmidomycin action in Plasmodium falciparum, in which a second potential target of the drug, IspD (the next enzyme in the pathway), was seen (34). No evidence of a second target was seen in our assay, although the lack of coverage of the pathway means that inhibition of IspD would not have been detected.

### (iii) CHIR-090.

After treatment with CHIR-090, an inhibitor of LpxC in lipid A biosynthesis (35), there were increases of several lipids annotated through IDEOM and decreases in others (see Data Set S2 in the supplemental material). Since the chromatography method used for the primary analysis was not able to adequately separate lipids, a secondary analysis was performed using a C_30_ column. This method provided better lipid separation and annotation, and analysis through LipidSearch revealed a large increase in lysophosphatidylethanolamine (LPE) and lysophosphatidylglycerol species (LPG) (Fig. 4b; see Data Set S7 in the supplemental material). The reaction preceding LpxC in lipid A biosynthesis is a reversible acyl transfer from acyl-ACP to UDP-*N*-acetylglucosamine (36). This enzyme has been shown to have an equilibrium constant that favors the reverse direction, i.e., the formation of acyl-ACP, and therefore the metabolic backup of the LxpC substrate could result in the production of more acyl-ACP, which may alter the flux of other lipid metabolites (37). The exact target of CHIR-090 was not identified using metabolomics, but the area of metabolism affected (lipid metabolism) was pinpointed, which provides additional information to the radioactivity assay.

### (iv) Drug modification.

**FIG 5:**
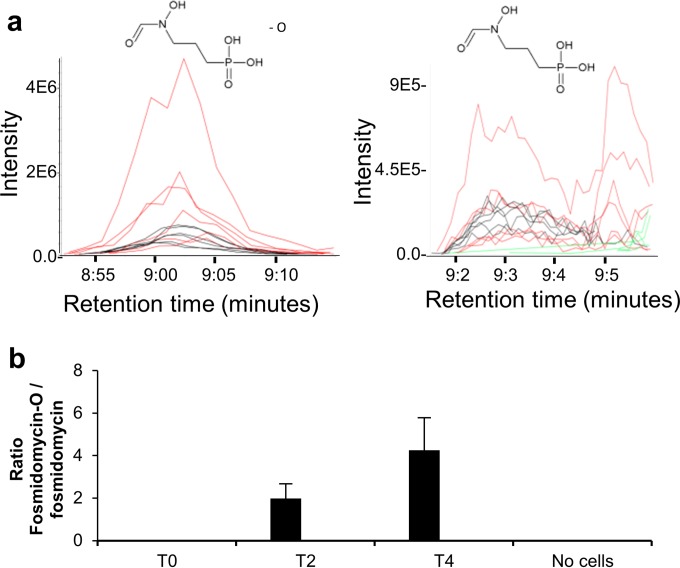
Fosmidomycin had a metabolite that appeared to be a cell-derived alteration of the drug. (a) Extracted peaks of deoxyfosmidomycin (left) and fosmidomycin (right). Black lines show the metabolite intensity at 2 h for each replicate, red lines show the intensity at 4 h, and green lines show the intensity in the 0-h control. (b) The ratio of deoxyfosmidomycin (fosmidomycin-O) to fosmidomycin increases during incubation with the cells. Error bars show standard deviations of the mean.

Modifications of AZ1 and CHIR-090 were not seen, but a metabolite consistent with the loss of an oxygen from fosmidomycin was seen in the fosmidomycin-treated samples (Fig. 5a; see Data Set S2 in the supplemental material). This deoxyfosmidomycin metabolite increased in the cell sample in a time-dependent manner but not in the absence of cells (Fig. 5b). Loss of oxygen could represent reduction of the hydroxamate group from fosmidomycin. Reduction of hydroxamate drugs *in vivo* has been reported previously (38), although future work is needed to ascertain the structure of this fosmidomycin metabolite and whether it possesses inhibitory activity.

### (v) Ceftazidime.

**FIG 6:**
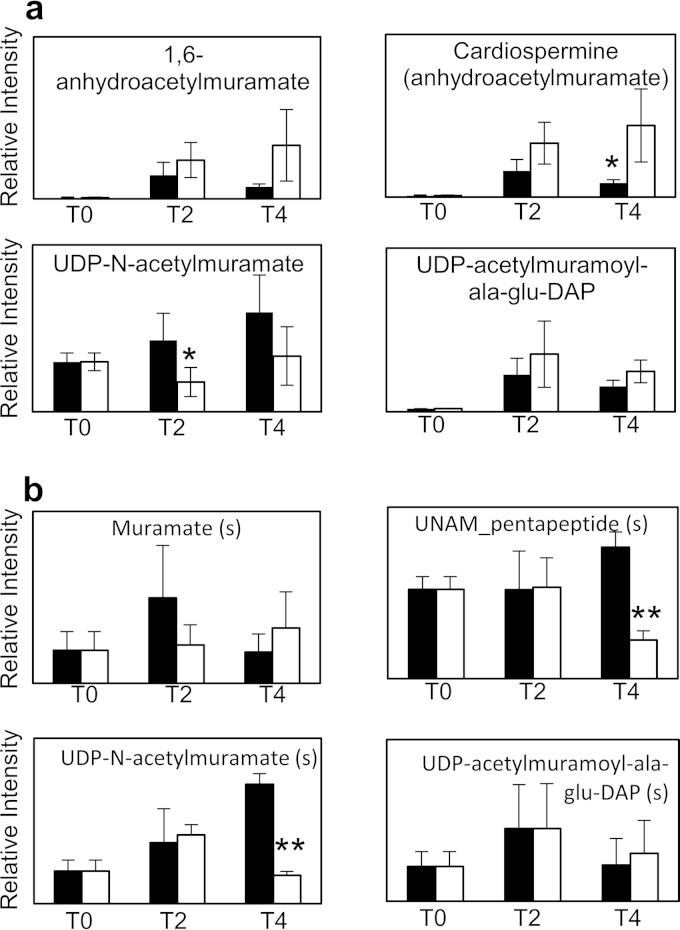
Changes to UDP-*N*-acetylmuramate and related peptides after ceftazidime treatment. (A) Changes after treatment with 4× MIC ceftazidime; (B) changes after treatment with 8×MIC ceftazidime. *, *P* value of <0.05; **, *P* value of <0.001 (Student's *t* test). “(s)” after the metabolite name indicates a match (mass and retention time) to an authentic standard. Black bars, no-drug control; white bars, treated with ceftazidime. Error bars show standard deviations of the mean.

Ceftazidime is a third-generation cephalosporin which works by inhibiting the cross-linking of peptidoglycan in the bacterial cell wall. Intact and cross-linked peptidoglycan molecules are too large to be detected using a metabolomics platform. Changes to the levels of metabolites earlier in cell wall biosynthesis were identified with a dose of 4× MIC (Fig. 6a), but these changes were less pronounced than changes to other metabolites not involved in cell wall biosynthesis (see Data Set S1 in the supplemental material). A higher dose of ceftazidime (8× MIC) was also unable to induce pronounced changes to these metabolites (Fig. 6b; see Fig. S1c and Data Set S5 in the supplemental material). Turnover of peptidoglycan has been shown to increase after beta-lactam antibiotic treatment in E. coli (39), and the small increases in these metabolic intermediates may be an indication of futile recycling of peptidoglycan in responses to cell wall disruption.

### (vi) 2-(Cyclobutylmethoxy)-5′-deoxyadenosine.

The pathway inhibited by 2-(cyclobutylmethoxy)-5′-deoxyadenosine, a DNA ligase inhibitor, could not be determined from the 4-h time course, as large areas of metabolism were affected (see Data Set S2 in the supplemental material). The untargeted analysis did identify significant disruption in DNA metabolism, however, when a shorter time course over 30 min was used, revealing large increases in purines, pyrimidines, and related metabolites (see Data Set S4 in the supplemental material). A large increase (on the order of 500-fold) in mass consistent with γ-glutamylputrescine was also detected after 2-(cyclobutylmethoxy)-5′-deoxyadenosine treatment over 4 h. As polyamines are not well separated on a pHILIC column, the samples were rerun on a HILIC column (see Data Set S6 in the supplemental material), and the polyamine pathway was analyzed in more detail. In addition to the large increase in γ-glutamylputrescine, there were smaller increases in masses consistent with diacetylspermine (8-fold) and diacetylspermidine (4.7-fold) but not in other metabolites of the polyamine pathway. These changes were not detected in untreated cells or in fosmidomycin-treated cells. The reason for this increase in acetylated or other modified polyamines is unknown, but polyamines have been shown to be produced in the presence of bactericidal agents as a response to oxidative stress (40).

### (vii) CCCP.

Carbonyl cyanide *m*-chlorophenylhydrazone (CCCP) had no measurable effect on the metabolome when the background variation was taken into account (see Data Set S3 in the supplemental material). CCCP inhibits oxidative phosphorylation by uncoupling the proton gradient in the electron transport chain and would be predicted to have pleiotropic effects on cellular pathways. A direct effect from CCCP exposure would be alteration of the adenylate energy charge (AEC); however, it is challenging to employ mass spectrometry-based measurements for the calculation of AEC due to the differences in the ionizability of the mono-, di-, and triphosphates. An additional explanation for the lack of perturbations seen with CCCP exposure is that the disruption of membrane translocation of proteins does not produce a distinct metabolomic signature (41).

### (viii) Triclosan.

Treatment with triclosan, an inhibitor of enoyl-acyl carrier protein reductase when bound with NAD^+^, produced more than 100 detected metabolite features with significant (*P* < 0.05) changes of >5-fold (see Data Set S3 in the supplemental material). Many of these changes related to an increase in masses annotated as glycerophospholipids. There were also decreases in masses consistent with methylthioadenosine, *S*-adenosylmethionine (matches authentic standard), and methylerythritol cyclodiphosphate. *S*-Adenosylmethionine is present in both the *S*-adenosylmethionine (SAM) cycle and in polyamine biosynthesis. The metabolites of both of these pathways were analyzed, and there was found to be a significant reduction in the majority of metabolites in polyamine biosynthesis but not in the SAM cycle. Glutathione biosynthesis was also severely affected by triclosan treatment, which, together with the changes in polyamine metabolism, suggests that the cells are suffering from oxidative stress. Polyamines interact electrostatically with negatively charged biomolecules, such as DNA, RNA, lipids, and acidic proteins (42). A lack of polyamines (putrescine, spermidine, and spermine) may therefore make cell membranes and DNA more susceptible to damage caused by oxidative stress. Adding a surplus (1 mM) of methionine, arginine, ornithine, putrescine, *S*-adenosylmethionine, glutathione, or cysteine did not rescue the cells from the effects of triclosan (MIC data not shown). The actions of triclosan on fatty acid metabolism may be compounded by an increase in oxidative stress.

A multifactorial mode of action at high doses for triclosan has been debated (43), and the polyamine changes seen here may relate to a membrane-destabilizing effect similar to those seen by Villalaín and colleagues (44).

### (ix) AZ7.

**FIG 7:**
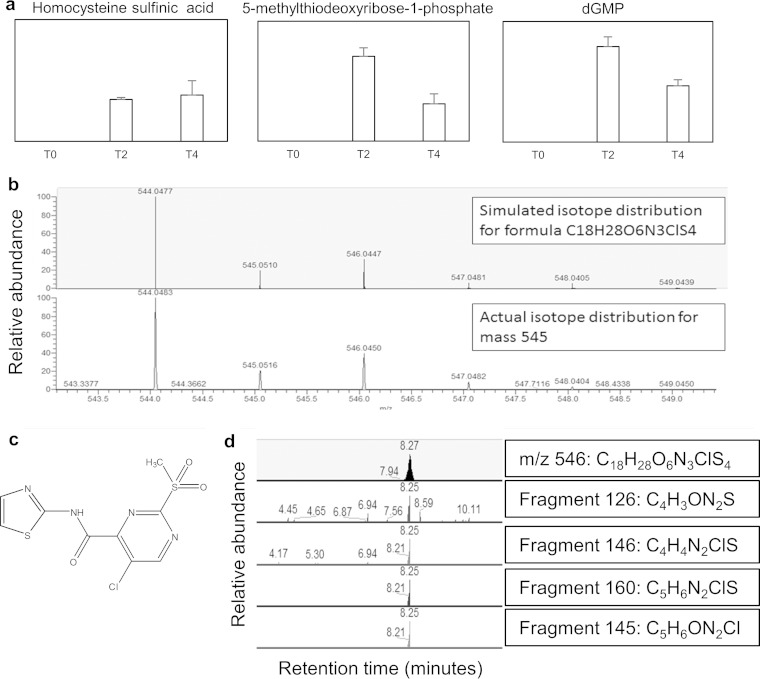
AZ7 causes alterations in sulfur metabolism. (a) Changes in sulfur metabolites after AZ7 treatment; (b) isotopic distribution of *m/z* 545.0551; (c) structure of AZ7; (d) fragments of *m/z* 545.0551. Error bars show standard deviations of the mean.

AZ7 has an unknown mode of action, and the radioactive precursor assay did not predict a specific area of inhibited metabolism (Table 1). There were many annotated metabolites altered after AZ7 treatment, including large increases in putative homocysteine sulfinic acid, methylthioribose phosphate, and dGMP (Fig. 7a). There were also several peaks present in only the drug-treated samples that could not be identified by using any of the databases incorporated into IDEOM. One of these peaks (neutral mass, 545.0551; retention time, 8.24 min) had a chlorine isotope pattern (Fig. 7b), indicating that it could be related to the drug AZ7, which also contains chlorine (Fig. 7c). The predicted formula of the peak *m/z* 545.0551 is C_18_H_28_O_6_N_3_ClS_4_.

Fragmentation of this *m/z* 545 revealed several fragments coeluting with the parent ion (Fig. 7d). Some of the fragments were predicted to contain chlorine, but analysis of the fragmentation data with mzCloud (https://www.mzcloud.org/; August 2015) produced no hits. Fragments *m/z* 126 and *m/z* 146 were also found in another mass that had a chlorine isotope pattern at retention time 6.94 min (Fig. 7d). This second mass (neutral mass, 925.1202) was also present in drug-treated cells but not in untreated cells or in the drug sample alone and had a predicted formula of C_29_H_48_O_13_N_10_ClS_3_.

To determine whether the masses seen were specific to AZ7 treatment of E. coli, human embryonic kidney cells were treated with the drug. *m/z* 545 was detected in HEK cells, but *m/z* 927 was not. Other masses with a chlorine isotope pattern were, however, found in HEK cells, suggesting that this conjugation of the chlorine-containing drug to intracellular metabolites is not specific to E. coli or to particular metabolites. In addition to its antibacterial activity, compound AZ7 also weakly inhibited growth of mammalian cells and may exert its effects through conjugation to sulfur-containing intracellular compounds. Knowing the mode of action of AZ7 may help to refine its structure to reduce toxicity.

### Conclusions.

The data presented here show that it is possible to use untargeted metabolomics to identify drug mode of action where drugs specifically target metabolic pathways. Metabolite extraction is simple and rapid, and a pipeline can be set up to treat cells with any compound, extract the metabolome, run the metabolome on a mass spectrometer, and analyze the data in an automated way. For some drugs, follow-up analysis may be required, either using different drug doses or treatment times or using a different column and mass spectrometer to analyze a more specific area of metabolism. Although 4× MIC appears to be a good starting dose for high-throughput analyses, it may be too high for some compounds inducing wide toxicity within the metabolome [as was seen with 2-(cyclobutylmethoxy)-5′-deoxyadenosine]. The dose of 4× MIC appeared to be too low to measure effects on cell wall metabolites from ceftazidime treatment, and a higher drug dose of 8× MIC was required. These kinds of modifications to the drug dosing may be required if the effects on internal metabolism are too great or too small, but 4× MIC remains a good starting concentration for higher-throughput assays.

A drug dose of 4× MIC appeared to be adequate to reduce E. coli growth over a 4-h time course for all seven drugs tested here. Time points of 0, 2, and 4 h were sufficient for many of the drugs under test, but a shorter treatment time was needed for 2-(cyclobutylmethoxy)-5′-deoxyadenosine, as the effects on metabolism were too prominent at 2-h posttreatment. Effects on the untreated control were evident, highlighting the need for adequate controls in these types of experiment. Other methods used to categorize drugs into classes based on their mode of action use statistical techniques to compare them to drugs of known classes. The method presented here does not require comparison with drugs of a known class and can produce much higher resolution data, in one case identifying the exact enzyme inhibited. Data on toxicity can also be gathered, as was the case with AZ7, where clues potentially pointing to reasons why this compound has unacceptable levels of toxicity in mammalian cells were gathered.

Untargeted metabolomics techniques using a metabolite identification software package such as IDEOM (25) were able to identify the target pathway or area of metabolism affected by an unknown drug in around 50% of cases (Table 3), but an adequate level of literacy in biochemistry is required. When this technique is combined with other, more pathway-specific analyses and knowledge of the drug structure and drug analogues, then predicting the mode of action of a drug will be greatly simplified. As data from further metabolome studies of untreated and drug-treated bacteria are generated and analyzed, databases can be constructed with the metabolite changes seen when certain targets or areas of metabolism are inhibited, adding further power to metabolomics-based prediction of mode of action.

**TABLE 3:** Summary of the performance of our metabolomics assay with the radioactivity-based assay

Compound	Radioactivity assay	Metabolomics MOA
1-[3-Fluoro-4-(5-methyl-2,4-dioxo-pyrimidin-1-yl)phenyl]-3-[2-(trifluoromethyl)phenyl]urea (AZ1)	DNA	Thymidylate kinase
2-(Cyclobutylmethoxy)-5′-deoxyadenosine	Mixed	DNA
Triclosan	Fatty acid	Not found
Fosmidomycin	No inhibition	MEP/DOXP pathway
CHIR-090	Mixed	Acyl lipids
CCCP	Mixed	Not found
5-Chloro-2-(methylsulfonyl)-*N*-(1,3-thiazol-2-yl)-4-pyrimidinecarboxamide (AZ7)	Mixed	Drug conjugation to S-containing metabolites?
Ceftazidime	Cell wall	Not found without knowledge of MOA

## Supplementary Material

Supplemental material
