# Supplementary material for: Untargeted Metabolomics To Ascertain Antibiotic Modes of Action
Source: Antimicrob Agents Chemother. 2016 Mar 25;60(4):2281–91. doi: 10.1128/AAC.02109-15 (PMC4808186; doi:10.1128/AAC.02109-15)
Supplement: Supplemental material [file supp_60_4_2281__index.html]

Untargeted Metabolomics To Ascertain Antibiotic Modes of Action — Supplemental material 

# Untargeted Metabolomics To Ascertain Antibiotic Modes of Action

## Supplemental material

- Supplemental file 1 -

  Supplemental Figure S1

  PDF, 529K
- Supplemental file 2 -

  Data Set S1: AZ1\_CAZ. IDEOM (see reference 25 in the main text) file with comparisons between 4X MIC of AZ1 (1-[3-fluoro-4-(5-methyl-2,4-dioxo-pyrimidin-1-yl)phenyl]-3-[2-(trifluoromethyl)phenyl]urea) and CAZ (ceftazidime) and the no-drug control (NDR) over 4 h. All metabolite names are putative annotations and should not be taken as the true identity of a metabolite.

  XLSB, 12M
- Supplemental file 3 -

  Data Set S2: AZ245. IDEOM (25) file with comparisons between 4X MIC of AZ2 [2-(cyclobutylmethoxy)-5'-deoxyadenosine], AZ4 (fosmidomycin), and AZ5 (CHIR-090) and the no-drug control (NDR) over 4 h. All metabolite names are putative annotations and should not be taken as the true identity of a metabolite.

  XLSB, 17M
- Supplemental file 4 -

  Data Set S3: AZ367. IDEOM (25) file with comparisons between 4X MIC of AZ3 (triclosan), AZ6 (CCCP), and AZ7 [5-chloro-2-(methylsulfonyl)-*N*-(1,3-thiazol-2-yl)-4-pyrimidinecarboxamide] over 4 h. All metabolite names are putative annotations and should not be taken as the true identity of a metabolite.

  XLSB, 16M
- Supplemental file 5 -

  Data Set S4: AZ2short. IDEOM (25) file with comparisons between 4X MIC of AZ2 [2-(cyclobutylmethoxy)-5'-deoxyadenosine] and untreated cells over 30 min. All metabolite names are putative annotations and should not be taken as the true identity of a metabolite.

  XLSB, 12M
- Supplemental file 6 -

  Data Set S5: 8xCAZ. IDEOM (25) file with comparisons between 8X MIC of CAZ (ceftazidime) and untreated cells over 4 h. All metabolite names are putative annotations and should not be taken as the true identity of a metabolite.

  XLSB, 9.8M
- Supplemental file 7 -

  Data Set S6: AZ24HILIC. IDEOM (25) file with comparisons between 4X MIC of AZ2 [2-(cyclobutylmethoxy)-5'-deoxyadenosine] and AZ4 (fosmidomycin) over 4 h. All metabolite names are putative annotations and should not be taken as the true identity of a metabolite.

  XLSB, 13M
- Supplemental file 8 -

  Data Set S7: AZ5lipids. LipidSearch output with comparisons between 4X MIC of AZ5 (CHIR-090) and untreated cells over 4 h.

  XLSX, 133K
