# Supplementary material for: Untargeted Metabolomics To Ascertain Antibiotic Modes of Action
Source: Antimicrob Agents Chemother. 2016 Mar 25;60(4):2281–91. doi: 10.1128/AAC.02109-15 (PMC4808186; doi:10.1128/AAC.02109-15)
Supplement: Supplemental material [file AAC.02109-15_zac004165040so1.pdf]

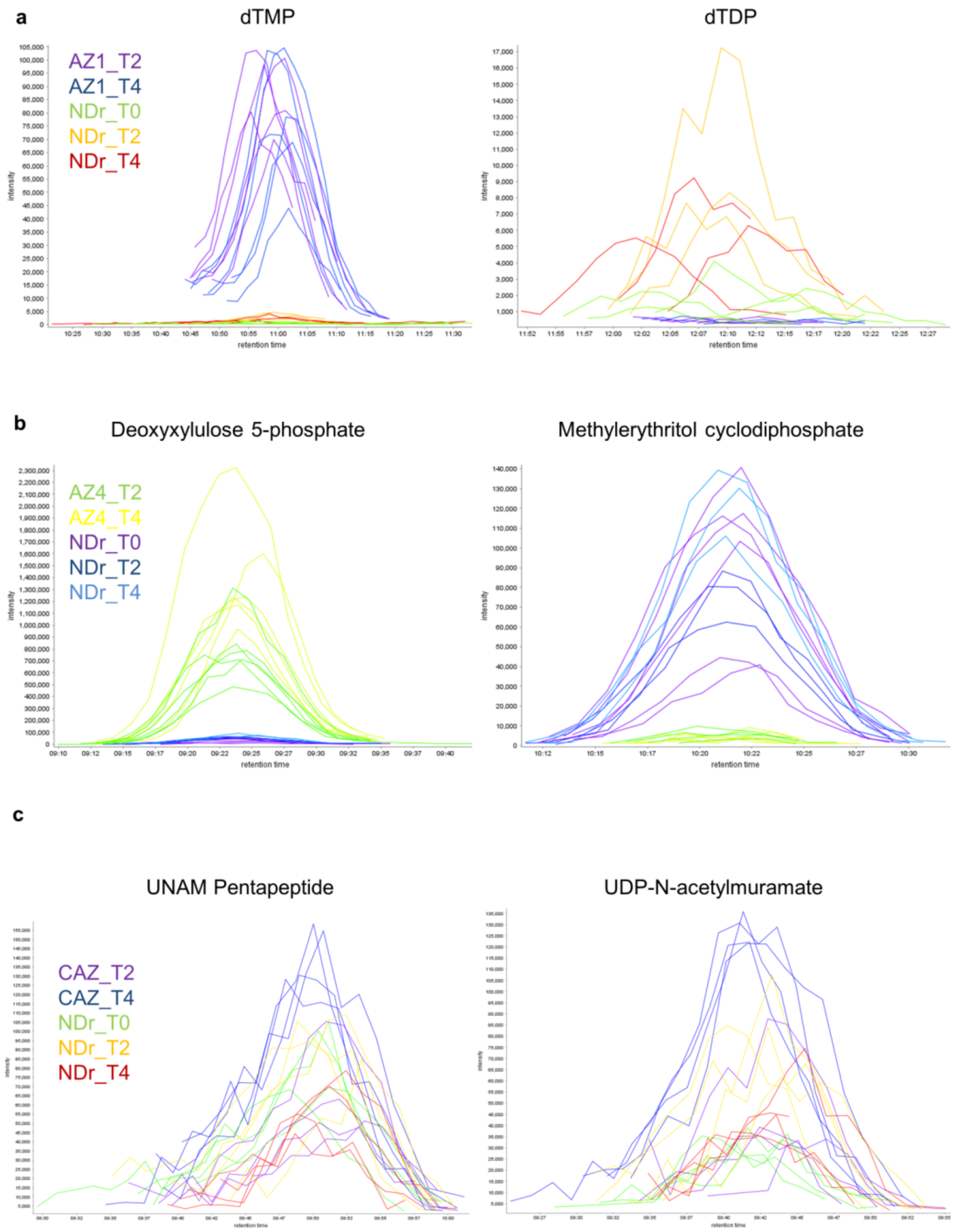

Supplementary Figure 1. Total ion chromatograms of selected metabolites. a) Chromatograms for dTMP and dTDP after treatment with 4xMIC AZ1, b) chromatograms for deoxyxylulose 5-phosphate and methylerythritol cyclodiphosphate after treatment with 4xMIC fosmidomycin, c) chromatograms for UDP-N-acetylmuramate and UNAM pentapeptide after 8xMIC ceftazidime.
